# The Atlantic Bonito (*Sarda sarda,* Bloch 1793) Transcriptome and Detection of Differential Expression during Larvae Development

**DOI:** 10.1371/journal.pone.0087744

**Published:** 2014-02-04

**Authors:** Elena Sarropoulou, Hooman K. Moghadam, Nikos Papandroulakis, Fernando De la Gándara, Aurelio Ortega Garcia, Pavlos Makridis

**Affiliations:** 1 Institute of Marine Biology, Biotechnology and Aquaculture, Hellenic Centre for Marine Research, Heraklion, Greece; 2 Instituto Español de Oceanografia (IEO), Centro Oceanografico de Murcia, Carretera de La Azohia, Puerto de Mazarron, Spain; National University of Singapore, Singapore

## Abstract

The Atlantic bonito (*Sarda sarda*, Bloch 1793) belongs to the important marine fish species with a wide geographical distribution covering the Atlantic Ocean, the Mediterranean and its bordering seas. Aquaculture practices for this species are still in their infancies and scientific studies are seldom undertaken, mainly because of difficulties in sampling. Thus for small tuna species like the Atlantic bonito only little is known about its biology and regarding the molecular background even less information is available. In the production of marine fish it is known that the most critical period is the larval stages, as high growth rates as well as significant developmental changes take place. In this study we have investigated the transcriptome of the Atlantic bonito of five larvae stages applying Illumina sequencing technology. For non-model species like aquaculture species, transcriptome analysis of RNA samples from individuals using Illumina sequencing technology is technically efficient and cost effective. In the present study a total number of 169,326,711 paired-end reads with a read length of 100 base pairs were generated resulting in a reference transcriptome of 68,220 contigs with an average length of 2054 base pairs. For differential expression analyses single end reads were obtained from different developmental stages and mapped to the constructed reference transcriptome. Differential expression analyses revealed in total 18,657 differentially expressed transcripts and were assigned to five distinguished groups. Each of the five clusters shows stage specific gene expression. We present for the first time in the Atlantic bonito an extensive RNA-Seq based characterization of its transcriptome as well as significant information on differential expression among five developmental larvae stages. The generated transcripts, including SNP and microsatellite information for candidate molecular markers and gene expression information will be a valuable resource for future genetic and molecular studies.

## Introduction

The Atlantic bonito (*Sarda sarda,* Bloch 1793), belonging to the family of the Scombridae is a mackerel-like epipelagic teleost fish species. Among the four allopatric species, *Sarda australis*, *Sarda chiliensis*, *Sarda orientalis* and *S. sarda*, only *S. sarda* inhabits the Atlantic and Mediterranean waters. Overall the Atlantic bonito has a wide geographical distribution covering the Atlantic Ocean, the Mediterranean and its bordering seas [1], [2]. It is an important marine fish species throughout its extent of distribution and is primarily exploited by coastal fisheries. In 2010 the total catch of Atlantic bonito was around 15,000 tons. The first successful aquaculture station was reported in 2011 within the European SELFDOTT project. For the production of marine fish, larval stages constitute a significant phase [3] as high growth rates as well as important developmental changes occur. Biotic as well as abiotic conditions influences greatly early stages of marine fish concerning survival, the start of feeding as well as larval growth. Phenotypic characters used for identification comprises the yolk shape, position of oil globule (if present), number of myomers, position of anus, fin fold and melanophores [4]. Concerning development of bonito species only a description of the larval growth in the Pacific bonito *S. chiliensis* (Cuvier) has been published [5]. The authors describe six basic stages of post-embryonic development based on ontogenetic changes in morphology and behavior. In addition two discrete metamorphic events during development are described with the first one being during the pre-juvenile stage and the second when the animals enter the juvenile stage [6]. To attain high quality juveniles and thus to assure the accessibility of healthy and well-developed juveniles the larval period is decisive. At molecular level some studies have been carried out in other fish species in order to assess differential gene expression of the egg and the early embryo [3], [7]–[13]. Post-embryonic larvae transcriptome assessment was addressed e.g. in the gilthead sea bream (*Sparus aurata*) by GS454 FLX sequencing [13] in order to get insight into its larval development. Among gene expression studies in early development of fish, a set of transcripts were identified showing specific gene expression patterns [14]–[16] drawing attention to the function of early expressed genes. Nonetheless for small tuna species like the Atlantic bonito only little is known about the biology and regarding the molecular background even less information is available. The fast evolving sequencing technologies facilitated the investigation in a large variety of different sequencing projects in model but also in non-model organism. Today a vast amount of sequence data are available for many species including non-model fish species of commercial interest like the catfish (*Ictalurus punctatus*) [17], [18], the gilthead sea bream *S. aurata* [13], [19] and the European sea bass (*Dicentrarchus labrax*) [20], [21]. However to the best of our knowledge, for economic epipelagic fish species like the Atlantic tuna or the Atlantic bonito only little genome and transcriptome information is published [1], [2], [22]. For the Atlantic bluefin tuna (*Thunnus thynnus*) in total 11, 453 sequences are published comprising 10,175 expressed sequence tags (ESTs) and 1278 Nucleotide (NCBI, July 2012). Regarding the Atlantic bonito even less molecular information is available. Only 469 sequences are published at the NCBI nr database and none in the NCBI EST database and no Next Generation Sequencing (NGS) project has been published so far (NCBI, November 2013).

The aim of the present study was to assess the transcriptome and the gene expression profile of five developmental stages of the Atlantic bonito ranging from the pre-larvae up to the juvenile. The detection of a great variety of transcripts and possible isoforms isolated for the first time in the Atlantic bonito are described. Furthermore the expression profiles for the five developmental stages with stage specific gene expression are discussed including part of the muscle transcriptome showing significant differential gene expression at the later stages.

## Methods

All procedures involving the handling and treatment of fish used during this study were approved by the HCMR Institutional Animal care and use committee following the three Rs (3 Rs, Replacement, Reduction, Refinement) guiding principles for more ethical use of animals in testing, first described by Russell and Burch in 1959. These principles are now followed in many testing establishments worldwide prior to initiation of experiments. The larvae were anaesthetized using 100–200 mg/L MS222 (tricaine methanesulfonate, Sigma-Aldrich, USA) depending on fish size. Afterwards samples were immersed in the RNA later (Ambion, Austin, TX, USA) were transferred to an 80°C ultra-low freezer until preparation of RNA. All experiments were carried out within the specific European RTD program of Framework Program 7, Theme 2-Food, Agriculture, Fisheries and Biotechnology (Project SELFDOTT) which is supervised by the European commission and follows the European Union Directive for the for the protection of animals used for experimental and other scientific purposes.

### Larval Rearing

Larval rearing was performed at HCMR and IEO following semi-intensive methodologies [23], [24] for a period of 40 days. Tanks were filled with natural seawater (salinity 40 ppt), while water for subsequent renewal was pumped from a littoral well (salinity 35 ppt, temperature 20±1°C).

### Sampling

**Figure 1:**
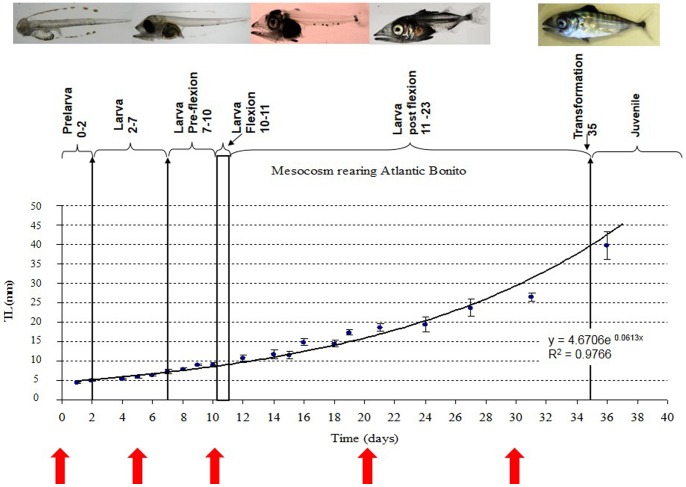
Developmental stages of *Sarda sarda*. Graphical presentation of Atlantic bonito developmental stages. Red arrows indicate sampling points. X-axis: days after hatching Y-axis: total length in mm.

In total three larvae (whole larvae) of five different developmental stages (0 dph, 5 dph, 10 dph, 20 dph and 30 dph) were pooled, collected in RNAlater (Qiagen, Hilden, Germany) and stored at −80°C for transcriptome analysis. Sampling points and maturation are illustrated in Figure 1. At the first stage, immediately after hatching, the larvae are almost colorless, they are feeding endogenously and have not developed sensing and digestive system (pre-larvae). At the second stage (5 dph) larval have developed a pigmented retinal epithelium, the lower jaw protrudes and melanophores are appearing on the dorsal surface, they are feeding exogenous food and have developed (although not fully) sensing and digestive systems. At the third stage (10 dph, flexion) the moving capacity of the individuals is improved allowing rapid acceleration towards prey and the digestive system is fully developed. The fourth (20 dph, post-flexion) stage is marked by increased melanophore pigmentation and further improvement of the swimming capacity, while the last stage studied (30 dph) is prior to the second metamorphic transition and represents the fully developed larvae (Figure 1).

### RNA Extraction

Whole larvae of each stage were submitted to RNA extraction. Disruption of the samples was performed in liquid nitrogen using mortar and pestle. After adding lysate buffer, the lysate was homogenized by passing it through a 20-gauge (0.9 mm) needle attached to a sterile plastic syringe for 5 times. RNA was subsequently extracted using the RNA extraction Kit II of Machinery Nagel (Dueren, Germany), according to the manufacturers’ instructions. RNA concentrations were determined using NanoDrop ND-1000 spectrophotometer (NanoDrop Technologies Inc., Wilmington USA) and the quality was assessed by electrophoresis on a 1% ethidium bromide agarose gel as well as by the A260/280 ratio and by Agilent 2100 Bioanalyzer using a RNA Nano Bioanalysis chip.

### NGS Sequencing

Samples for RNA sequencing (RNA-Seq) were prepared by Cornell University Core Laboratories Center using standard methods and sequenced over two lanes of Illumina HiSeq vs 2000. In one lane, extracted RNA from mixed developmental stages were sequenced as paired-end, 100 bp reads. On the second lane, each of the five developmental stages were tagged and sequenced as 100 bp, single-end reads. Reads from each stage were distinguished through the use of mulitplex identifier (MID) tags. Sequence quality was assessed using FastQC (version 0.10.0; http://www.bioinformatics.babraham.ac.uk/projects/fastqc) and low quality reads were removed with Trimmomatic software [25]. Sequence data from the mixed developmental stages were assembled using Trinity, version 2012-06-08 [26]. Raw sequence data are submitted to the Short Read Archive (SRA) database of NCBI under the accession numbers SAMN02044484 (Mixed stages), SAMN02044485 (Bon0), SAMN02044486 (Bon5), SAMN02044487 (Bon10), SAMN02044488 (Bon20), SAMN02044489 (Bon30).

### Single Nucleotide Polymorphism (SNP) and Microsatellite Identification

Sequences were clustered using the default parameters of the CD-HIT-EST tool [27] and only the longest transcripts were retained. The sequence alignment data of these transcripts where then screened for sites with possible single nucleotide polymorphic (SNP) variation as well as patterns of simple sequence repeats (SSR) using freebayes (version 0.8.7; http://bioinformatics.bc.edu/marthlab/FreeBayes) and Msat identification tool [28] respectively. SNPs were called when a site had a minimum coverage of 20 reads where at least 35% of the reads with quality scores greater than 30 supported the alternative allele. Microsatellites were reported for transcripts carrying motifs of di-, tri-, tetra-, penta- and hexa-nucleotides for a minimum of 8 repeats.

### Expression Analysis

Transcripts expression profiles were assessed for each developmental stage using the single read sequences retrieved from each stage by mapping the reads against the reconstructed assembly using bowtie [29] allowing maximum of 3 mismatches throughout the entire length of the read. Expression abundances were quantified using RSEM version 1.2.3 [30]. Contigs with low read support (less than one read per million mappable reads) were excluded from downstream analysis. Pairwise abundance of transcript estimates between any two developmental stages were investigated using the R Bioconductor package DESeq [31]. Contigs that in accordance to DESeq, were found to have a *p*<0.05 and a minimum of 2 fold differences in their expression between different stages were assigned as differentially expressed.

### Functional Annotations and Gene Ontology

Assembled transcripts were submitted against the non-redundant protein database (nr) as well as the non-redundant nucleotide database (nr/nt) using the standalone BLAST tools (version 2.2.25) [32] with cut-off e-values of 10^−6^ and 10^−10^ respectively. Annotations and GO terms were assigned using Blast2Go software [33].

### Cluster Analysis

Transcripts assigned to be differentially expressed were submitted to K-means clustering method. K means clustering is the most appropriate clustering method when more than 200 data points are analysed [34]. The cluster number was set from 3 to 10. Distances were computed using simple Euclidean distance and the maximum number of iteration was fixed to 50. The cluster-cluster distance was calculated by determining the distance between centroids. Discriminant analysis, testing the classification of groups obtained by K-means clustering was performed. For K-means clustering and Discriminant analysis the statistical software package SPSS 12.0 (SPSS; Chicago, IL) was used. Heatmaps of obtained clusters were constructed using the R-Package [35].

### VENN Diagram and Enrichment Analysis

Venn diagram was constructed using all differentially expressed genes using as reference stage Bon0. To find enrichment in gene ontology (GO) terms, enrichment analysis (Fisher’s Exact Test) tool in Blast2GO software was used with term filter value p<0.05, term filter mode “False Discovery Rate (FDR)” and two-tailed test. Reference data set were all mapped genes onto the constructed reference transcriptome and test data set used in the present analysis were all transcripts found to be differentially expressed only at stage 30 when comparing all stages under study to stage 0.

### Network Analysis

In order to infer the module networks the LeMoNe algorithm was used. LeMoNe uses ensemble based probabilistic optimization techniques to identify clusters of co-expressed transcripts as well as their regulators. In this study the normalized log2 values of all stages were used as input of transcript expression. Annotated transcription factors were used as potential regulators. LeMoNe assigned the corresponding regulators in each module characterized by a particular weight. The visualization of the regulatory networks was performed with Cytoscape v. 2.7.0, where transcripts being differentially expressed only between stage Bon0 and Bon5 were filtered out in the present study.

### Phylogenetic Tree Construction

Homologue nucleotide sequences of comp49187_c0_seq1 and comp6057_c0_seq1 with the blastx match myozenin-2-like (Table S1) were used for phylogenetic tree construction. Multiple sequence alignments were performed using ClustalW on Bioedit. Maximum likelihood analysis was performed using MEGA4 [36], [37] and the phylogenetic tree was obtained after burning 1,000 trees. The numbers at the nodes indicate posterior probability values. The tree was rooted using the myozenin-2-sequence from human and horse.

## Results

### Transcriptome Sequencing

RNA Seq was carried out on RNA extracted from 5 different developmental stages of the Atlantic bonito. A total number of 169,326,711 paired-end reads with a read length of 100 bp were generated. After filtering 162,109,074 clean reads were obtained for assembly analysis (Table 1).

**Table 1:** Summary of Illumina reads used for transcriptome assembly retrieved from one library containing different developmental stages.

Illumina HiSeq2000 vs3 paired-end reads	
**Left read:**	
Total number of reads used for assembly:	162,109,074
Total number of base pairs:	16,039,836,009
Average number of read len:	98.94
**Right read:**	
Total number of reads used for assembly:	162,109,074
Total number of base pairs:	16,251,330,770
Average number of read len:	100.25
**Total number of reads:**	169,326,711
Both ends survived:	162,109,074 (95.74%)
Forward survived:	3,992,316 (2.36%)
Reverse survived:	1,881,149 (1.11%)
Dropped:	1,344,172 (0.79%)
**Assembly statistics after filtering**	
Number of contigs:	68,220
Total number of base pairs:	139,888,259
Number of bases per nucleotide type:	A: 37,741,180; C: 32,508,872; G: 32,639,932; T: 37,202,26
Average number of contig len:	2054
N50:	3011
N90:	1013

### *De novo* Assembly of the Atlantic Bonito Transcriptome

The assembly returned 68,220 contigs with an average length of 2054 bp (Table 1) and is submitted to the Fish-*it* database (http://www.fish-it.org). This set was used as reference transcriptome for differential expression assessment as well as for the enrichment analysis. Putative single nucleotide polymorphisms (SNPs) and microsatellites were identified and summarized in Table 2 and Table S2.

**Table 2:** Summary of putative SNP identification from the Atlantic bonito (*S. sarda*) expressed short reads.

	Atlantic bonito (*S. sarda*)
Contigs under analysis	55213
Total SNPs	156507
Transitions	23375
Transversions	133132
Total Microsatellites	2714
AC	784
AG	412
AT	226
CT	183
GT	1096
tri-nucleotide	CTC (2x), TGA
tetra-nucleotide	GATT(2X), TCCT,TGGC, ATTT, CAAA
Hexa nucleotide	TGGAGC (3X), CTAATG

Putative SNPs site include all base variation with a minimum of 20 reads and at least 35% of the reads support the alternative allele.

### Gene Identification and Annotation

**Figure 2:**
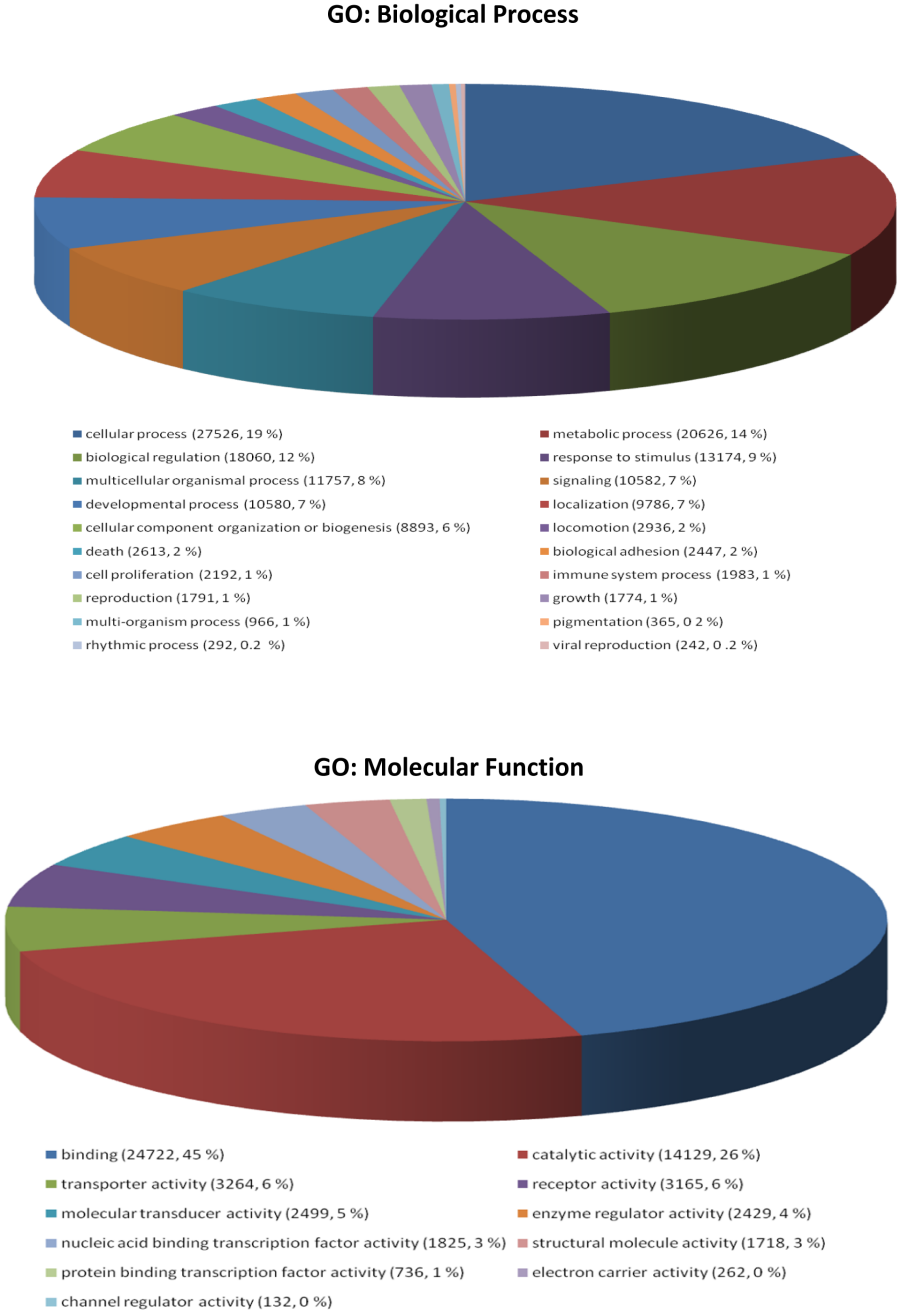
GO annotation. Distribution of GO annotation terms at level 2 for biological process and molecular function in the Atlantic bonito transcriptome.

BLASTX and BLASTN searches were performed for annotation of the Atlantic bonito transcriptome assembly. Out of 68,220 contigs obtained after assembly, 43,312 (∼63%) showed a significant match against the protein database (nr) and 46,224 (∼ 68%) had a positive match against the nucleotide database (nr/nt) of GenBank. Blast2GO analysis of the different annotations related to biological process revealed that the majority of transcripts are categorized to nine gene ontology (GO) terms: Cellular process (19%), metabolic process (14%), biological regulation (12%), response to stimulus (9%), multicellular organismal process (8%), signaling (7%) developmental process (7%), localization (7%) and cellular component organization of biogenesis (6%). Blast2GO analysis of the different annotations related to molecular function classified the majority of transcripts to the binding (45%) and catalytic activity (26%) categories (Figure 2).

### Assessment of Differential Gene Expression

**Figure 3:**
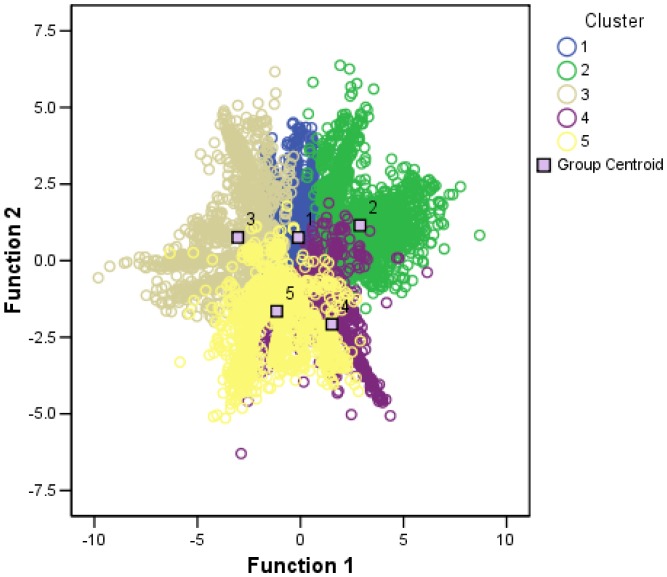
Canonical discrimination functions. Clustering of clones with differential expression identified by DESeq (pval <0.05) in each pair wise comparison. The data points given (cases 1–5) were grouped in five main clusters (colored yellow, grey, blue, green and violet respectively). The group centroid represents the average value of the cases contained in each cluster.

**Figure 4:**
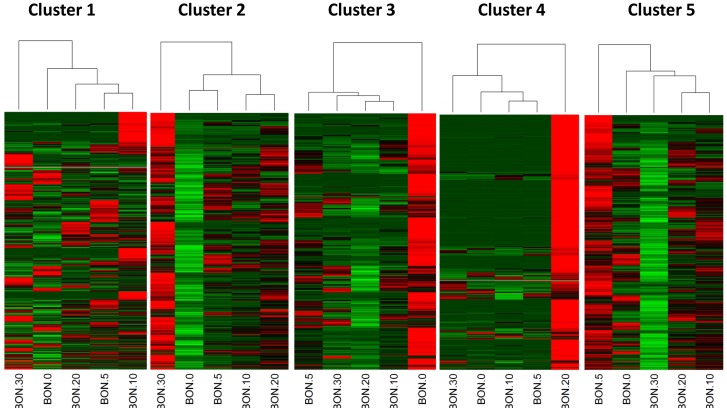
Illustration of the five clusters by heat map images. Heat map images showing the genes grouped by the K-means clustering method. Stages are indicated under each column. Gene expression is shown in rows. The quantitative changes in gene expression are represented in color: red indicates up-regulation whereas green indicates down-regulation.

Single end reads were assessed from each stage separately and mapped onto the constructed reference transcriptome (Table 3). On average 82% of the sequence reads from each stage were mapped successfully and used for downstream expression analysis. Expression abundances are shown in Table S1. In total 18,657 transcripts were found to be differentially expressed (*p* value <0.05, and minimum of 2 fold difference) in one of the pair-wise comparison of the stages. Those transcripts found to be differentially expressed at least in one of the stages, were subjected to K-means clustering having the largest F-values when the cluster number was set to five (data not shown) (Figure 3). Cluster methods are frequently used for grouping genes by their expression patterns. Expression profiles are visualized as heatmaps using the R software package (Figure 4). The color displays up (red) and down (green) regulation between the stages. Clearly each of the five clusters shows genes specifically expressed in one of the five stages. Discriminant analysis confirmed K means clustering method by successfully grouping 94.1% in the predicted clusters.

**Table 3:** Summary of mapping statistics obtained from five different developmental stages.

Sample	# of reads	# reads with at least one reported alignment
Bonito 0 dph	36,846,289	30,514,092 (82.81%)
		(single-end)
Bonito 5 dph	40,043,500	33,499,703 (83.66%)
		(single-end)
Bonito 10 dph	37,598,540	31,651,338 (84.18%)
		(single-end)
Bonito 20 dph	38,244,043	31,096,139 (81.31%)
		(single-end)
Bonito 30 dph	52,154,449	42,934,570 (82.32%)
		(single-end)
Mixed Stages	169,326,711	139,359,237 (82.30%)
		(paired-end)
**TOTAL**	**374,213,532**	

### Analysis of Differentially Expressed Genes

**Figure 5:**
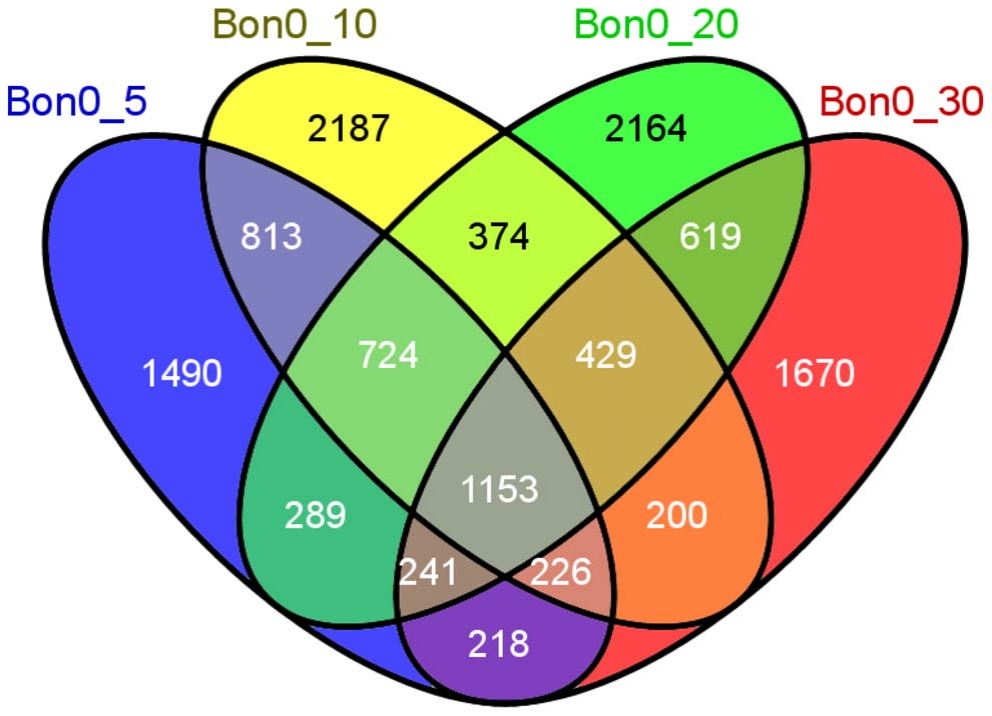
Venn diagram. Venn diagram comparing significant differentially expressed genes between stages 5

**Figure 6:**
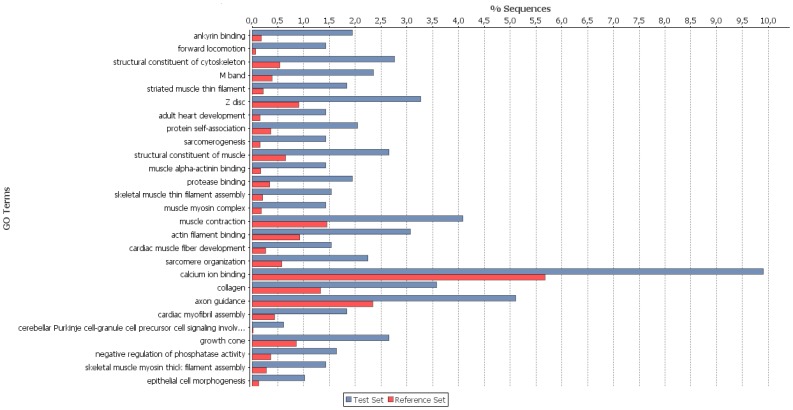
Differential GO-term distribution. Enrichment analysis with the set of genes found only in Atlantic bonito stage 30: transcripts obtained after assembly of mixed developmental stages. Test set: transcripts found only in Atlantic bonito stage 30 after comparison to stage 5, 10 and 20 dph.

**Figure 7:**
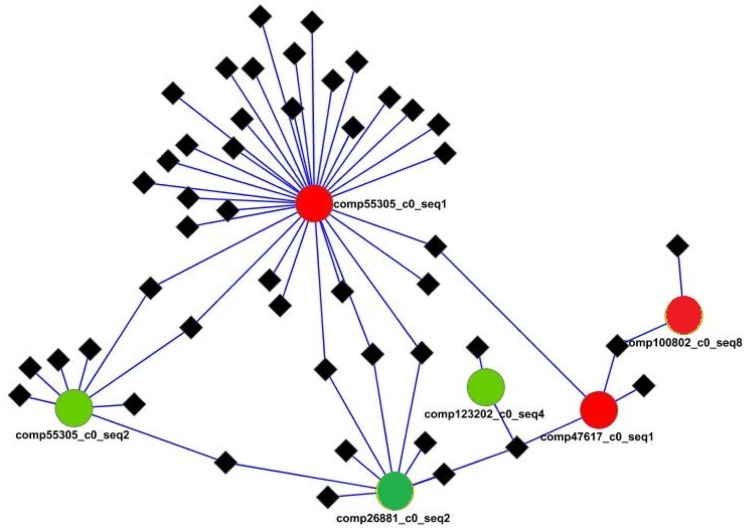
Network analysis. Simplified representation of the module network inferred by the LeMoNe algorithm. Clusters of co-expressed genes (modules) have diamond black shapes, while transcription factors are symbolized by circles. The color of the circle correspond either to up-regulation in stage Bon5 dph (red) or down-regulation (green) in stage Bon5 dph in relation to sage Bon0 dph. Blast matches of illustrated transcription factors are as followed: **comp55305_c0_seq1 and comp55305_c0_seq2**: general transcription factor IIH subunit 2-like; **comp26881_c0_seq2**: transcription factor ETV6-like; **comp47617_c0_seq1**: transcription factor HIVEP2; **comp123202_c0_seq4**: POU domain, class 2, transcription factor 1-like; **comp100802_c0_seq8**: transcription factor CP2-like protein 1.

**Figure 8:**
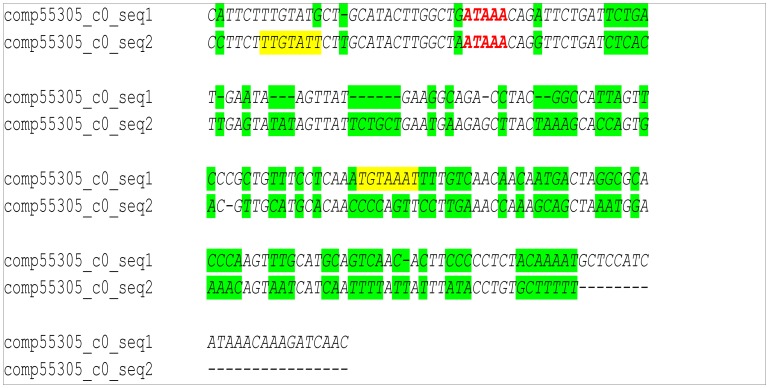
3′UTR alignment. Alignment of the end of the 3′UTR of two different forms of one transcript, general transcription factor IIH subunit 2-like (comp55305_c0_seq1 and comp55305_c0_seq2) identified as main hubs in the network inferred by the LeMoNe algorithm. The two transcripts differ from each other only at the end of the 3’UTR which comprises two conserved 7 mers (PUF, and CIF, Table 5) where the one is present in comp55305_c0_seq1 and the other in comp55305_c0_seq2. Both conserved 7 mers were retrieved from Andreassen et al. [34]. Complete alignment of the two transcripts is shown in Figure S1.

Stage specific transcripts using stage Bon0 as reference stage (in total here 12,797) are illustrated by a Venn diagram in Figure 5. The number of transcripts found in only one of the stages are within the same size range [on average 1878 (∼ 15%)], and 1153 (9%) transcripts are in common for all five stages. Enrichment analysis with the set of genes found uniquely in Atlantic bonito stage 30 dph and using all transcripts obtained after assembly of mixed developmental stages as reference test set revealed genes mainly involved in muscle development (Figure 6). All major sarcomeric proteins found are listed in Table S3. Investigation in transcripts appearing early in development is of special interest as major functional mechanisms are set. Several studies have shown that particular gene families like homeobox proteins, nuclear receptors, sox genes and forkhead box proteins have established roles in developmental processes. Table S4 summarizes all transcripts found in the present study with a relevant role in the post-embryonic development. Thus, after network analysis, the focus was primarily to investigate the relation of differentially expressed transcription factors between stages Bon0 and Bon5 (Figure 7). Interestingly two of the main hubs seem to be two different forms (comp55305_c0_seq1 and comp55305_c0_seq2) of one gene (general transcription factor IIH subunit 2-like). Those two transcripts differ from another only at the end of their 3’UTR (Figure 8, Figure S1). The third hub within the main network is coding for the transcription factor ETV6-like (comp26881_c0_seq2) which shows up regulation at stage 0 compared to stage 5. According to the KEGG pathway the transcription factor ETV6-like is involved in the dorso-ventral axis formation showing the importance of this transcript within development.

## Discussion

**Figure 9:**
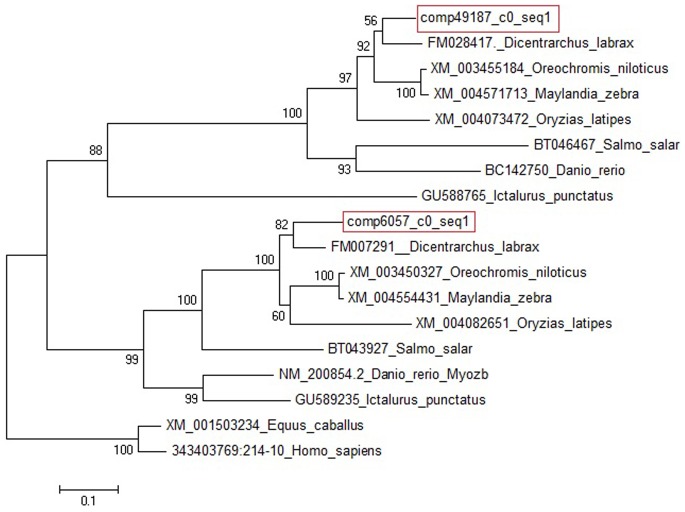
Molecular Phylogenetic analysis by Maximum Likelihood method. The phylogenetic tree of myozenin-2-like was inferred by using the Maximum Likelihood method based on the Tamura-Nei model [36]. The percentage of trees in which the associated taxa clustered together is shown next to the branches. The tree is drawn to scale, with branch lengths measured in the number of substitutions per site. The analysis involved 18 nucleotide sequences. Codon positions included were 1st+2nd+3rd+Noncoding. There were a total of 886 positions in the final dataset. Evolutionary analyses were conducted in MEGA5 [37].

This work describes the first assessment of the Atlantic bonito transcriptome as well as differential expression between five developmental stages. During the production cycle of marine fish, the welfare of larval stages are of great importance as they experiences high grow rates and impressive changes in anatomy and physiology [3]. Several studies have shown that RNA Seq is a robust approach to perform transcriptome profiling [2]–[4]. Most studies in non-model fish species have used GS454 Titanium technology for RNAseq and assessment of differential expression. However in order to obtain high throughput several runs have to be performed (e.g. [19]) or/and differential expression can be detected by 3’ UTR tagging [21]. In the present study a reference transcriptome of the Atlantic bonito was constructed using paired end reads (PE) of one Illumina lane (∼ 16 Gb, Table 1) from equally mixed developmental stages. The paired-end reads generated resolve assembly problems due to repetitive regions. The number of contigs obtained after assembly are counted to 68,220 with an average length of 2054 bp and N50 of 3011 bp (Table 1). For non-model species high throughput 454 sequencing which provides longer reads has been widely used up until today as *de novo assembly* of short reads without a reference transcriptome were still difficult. However using Illumina technology of sequencing paired-end reads facilitates the assembly and using a combined assembly and mapping strategy results in a reasonable and accurate reference transcriptome which is also strongly supported by the obtained results of sequence average length and N50 length. Transcripts expression profiles were assessed by high throughput single end sequence (SE) data from each developmental stage which was mapped against the reconstructed assembly using bowtie [29] (Table 3). As for *de novo* transcriptome assembly PE reads are of importance, for assessing differentially expressed genes the sequencing depth is significant [38], [39], [40]. PE does not give more statistical power than SE as a PE read and a SE read both count as a single tag. However for differential expression analysis only counting tags is critical. Thus by performing an additional Illumine SE sequencing for each stage separately the acquired sequencing depth for detection of differential expression was assured. In addition the high mapping percentages of the single reads obtained from each stage (∼80%, Table 3) confirm the high and accurate transcriptome assembly. Nevertheless RNAseq using NGS technology has to be considered as a scan of the transcriptome in order to set the basis for further functional studies on specific genes. In the present study transcripts were annotated by performing BLASTX and BLASTN searches against the databases of NCBI. Several transcripts are showing similar or even the same BLAST matches (Tables S3 and S4). They may present paralogues or different isoforms. One example of identical gene blast match represents the match “PREDICTED: myozenin-2-like [*Oreochromis niloticus*]” of the two transcripts comp49187_c0_seq1 and comp6057_c0_seq1. Here homolog sequences for seven fish species were retrieved from NCBI databases followed by phylogenetic tree construction (Figure 9). Two clearly separated groups were identified characterizing the one group as *myozenin 2b* and the other as *myozenin 2* or *myozenin-like* but not as *myozenin 2a*. Thus here most probably an additional paralogue exists. All transcripts with similar or identical BLAST match were aligned to each other to assure their uniqueness. In addition comparative BLAT search were performed to four model fish species mapping some of those transcripts to different chromosomes (Table 4). This confirms the existence two transcripts with the same or similar BLAST matches and pinpoints to possible paralogues genes.

**Table 4:** Comparative mapping of transcript with same or similar blast match to four model fish species with available whole genome sequences.

Blast match	Transcript	*Danio rerio*	*Oryzias latipes*	*Gasterosteus aceculeatus*	*Tetraodon nigroviridis*
homeobox protein otx5-like	comp11286_c0_seq2	15	14	VII	7
	comp11286_c0_seq3	15	13	I	16
transcription factor SOX-6-like isoform 1	comp23068_c1_seq17	7	3	II	5
transcription factor Sox-6-like	comp23068_c1_seq15	7	6	XIX	5
	comp23068_c1_seq18	7	6	XIX	13
Sox 19	comp96498_c0_seq1	7	18	VII	Un
	comp72863_c0_seq1	n/a	n/a	VII	Un
Sox 11b	comp372390_c0_seq1	20	Scf 709	n/a	10
	comp29608_c2_seq1	20	22	XV	10
homeobox protein engrailed-2a-like	comp85602_c0_seq1	7	20	XXI	6
	comp85674_c0_seq1	7	20	XXI	6
homeobox protein engrailed-2b-like	comp204683_c0_seq1	n/a	n/a	III	Un
homeobox protein orthopedia B-like isoform 1	comp47586_c0_seq6	21	9	XIII	12
	comp55341_c0_seq2	21	12	XIV	4
	comp67761_c0_seq1	21	9	XIV	4
homeobox protein orthopedia B-like isoform 2	comp47586_c0_seq5	21	9	XIII	12
Zinc finger homeobox protein 4	comp36475_c0_seq8	24	20	XXI	6
	comp3751_c0_seq12	7	20	XXI	Un
nuclear receptor subfamily 1 group D member 1-like	comp31421_c0_seq1	23	5	Un	11
	comp31421_c0_seq2	23	5	n/a	11
	comp41077_c0_seq1	n/a	16	X	Un
Nuclear receptor subfamily 1 group Dmember 2-like	comp110302_c0_seq1	11	7	XII	9
	comp96775_c0_seq1	23	7	XII	
	comp50659_c0_seq1	n/a	5	Un	11
	comp6400_c0_seq3	19	scf3618	X	Un
	comp6400_c0_seq4	19	scf3618	X	Un
Myozenin-2-like	Comp49187_c0_seq1	14	10	IV	1
	Comp6057_c0_seq1	1	22	VII	Un
calpain-2	comp4444_c1_seq1	22	ultractg1	IX	n/a
	comp4444_c1_seq4	22	scf1395	IX	19
obscurin-like	comp67664_c0_seq38	8	4	VIII	2
obscurin-like protein 1-like	comp48590_c0_seq1	6	2	I	4
troponin I, slow skeletal muscle-like	comp17106_c0_seq1	18	n/a	XIX	20
	comp63836_c0_seq1	6	5	XVII	n/a

A common step for microarray analysis is cluster analysis of differentially expressed genes. Cluster analysis permits to classify samples of entities into a small number of mutually exclusive groups based on the similarities among the entries. In the present study the obtained groups were confirmed by discriminant analysis in which up to 95% were successfully grouped in the predicted clusters. Four of the five clusters show exclusive up-regulation in one of the developmental stages, i.e. cluster 2 stage Bon 30, cluster 3 stage Bon 0, cluster 4 stage Bon 20 and cluster 5 stage Bon 5. Cluster 1 comprises a small set of genes showing up-regulation exclusively in stage Bon 10 (Figure 4). Transcripts of each cluster including their annotations are listed in Table S1. In addition, genes belonging to gene families with established roles in embryonic and developmental processes as described by Yufera et al. [13] were identified to be differentially expressed between the five stages (Table S4). Interestingly looking at genes belonging to the homeobox protein family showed that they were present and up-regulated in clusters 1, 3 and 5 but absent or low abundant in cluster 2 and 4. The former three clusters comprise mainly genes up regulated at the earlier stages i.e. stage 0 dph (clusters 3 and 5) and stage 5 dph (clusters 1 and 5). Cluster 4, with only two representatives of the homeobox protein family mainly comprises transcripts found to be up-regulated at stage 20 dph and cluster 2 with no representative of the homeobox protein family comprises mainly transcripts found to be up-regulated at stage 30 dph. This clearly shows the significance of the first three stages in relation to development as homeobox proteins are known to play an important role in pattern formation. Furthermore homeobox proteins like orthodenticle-related (OTX), LIM and visual system which are involved in eye formation are also found to be up- regulated in the first three stages studied. Eye formation is of importance for pelagic fish as they have to hunt for feeding. In zebrafish it has been shown that OTX transcription factors are required for retinal pigment epithelium development but unlike in mouse microphthalmia-associated transcription factor (*Mitf*) is not [41]. In the present study *Mitf* has been identified but does not show significant differential expression within the five stages. In contrast to *Mitf* the role of *Otx* in eye formation is conserved between zebrafish and other vertebrates indicating it importance in development. Besides transcripts which show differential expression between stages also transcripts were found exclusively in only one of the stages. Figure 5 shows a Venn diagram where stage Bon0 was used as reference stage. Consequently transcripts being present only in one of the other stages can be filtered out. The amount of stage specific transcripts varies from 1,490 in stage 5 to 2,187 found to be present only in stage 10. By enrichment analysis using those transcripts found only in stage Bon30 revealed that the majority of them are involved in muscle development. These results are to be expected as animals in the late larvae post-flexion stage form the characteristic body shape for scombrids and develop lateral muscle fields. In addition the inactive periods of the previous stages are not present and animals are more or less constantly active. Table S3 shows all muscle genes found which were described in a previous study of fast skeletal muscle transcriptome of sea bream [19]. Besides the identification of important transcripts of the Atlantic bonito muscle transcriptome this result also corroborate the experimental set up and analysis method used in the present study as it is in accordance to the physiology of the larvae development. Interestingly, network analysis of differentially expressed transcripts between stage Bon0 and stage Bon5 revealed that two isoforms are differentially expressed with regard to each other (Figure 7). Whereas the one isoform of the general transcription factor IIH subunit 2-like (comp55305_c0_seq1) is up-regulated the other one (comp55305_c0_seq2) is down-regulated. The two different forms of the transcript transcription factor IIH subunit 2-like were identified as main hubs in the network inferred by the LeMoNe algorithm. The two transcripts differ from each other only at the end of the 3’UTR. However this region comprises two conserved 7 mers (PUF, and CFI, Table 5) where the one is present in comp55305_c0_seq1 and the other in comp55305_c0_seq2. Both conserved 7 mers were retrieved from Andreassen et al. [42] where authors describe in total 11 conserved 7 mers in the Atlantic salmon being significantly over-represented in the 3’UTR. The Puf family is known to bind target sequences in the 3′UTRs and in this manner to regulate mRNA expression whereas the CFI target sequence is in involved in polyadenylation site recognition.

**Table 5:** Conserved 7-mers found in comp55305.

Motif	Position	Found in sequence	Proteins known to bind3’UTR target sequence
TGTCTGT	658 bp	Comp55305_c0_seq1 and seq2	n/a
TGTCTGT	918 bp	Comp55305_c0_seq1 and seq2	n/a
TTGTATT	2859 bp	Comp55305_c0_seq1 and seq2	CFI
TTGTATT	2807 bp	Comp55305_c0_seq2	CFI
TGTAAAT	1545 bp	Comp55305_c0_seq1 and seq2	PUF
TGTAAAT	2918 bp	Comp55305_c0_seq1	PUF

## Conclusion

The present study is the first report of a transcriptome study in the Atlantic bonito (*S. sarda*). We demonstrate that transcriptome analysis as well as assessment of differential expression of RNA samples using Illumina sequencing technology is technically efficient and with low cost. A total of 68,220 contigs have been constructed out of a total number of 169,326,711 100 bp paired-end reads. Differential expression between five important developmental stages has been assessed and stage specific genes were isolated. It has been shown that in earlier stages transcripts like homoebox genes are up-regulated whereas in the later stage transcripts important for muscle development are found to be higher expressed. The generated transcripts, microsatellite and SNP information for candidate molecular markers as well as gene expression information will be valuable information for future genetic and molecular studies in the Atlantic bonito and closely related species pinpointing also to the importance of studying the 3’UTR as well as paralogues during development.

## Supporting Information

Figure S1 Supplemental figure to figure 8 in the manuscript. Figure 8 shows only part of the 3′UTR, which differs from each other. Here the whole alignment of the cds is shown including the start and the stop codon.
(DOCX)

Table S1 Transcripts of each of the five generated cluster including their annotations.
(XLSX)

Table S2 Transcripts screened for sites with possible single nucleotide polymorphic (SNP) variation as well as patterns of simple sequence repeats (SSR) using freebayes (version 0.8.7; http://bioinformatics.bc.edu/marthlab/FreeBayes).
(XLSX)

Table S3 Transcripts involved in muscle development. Here all major sarcomeric proteins are shown found after enrichment analysis using enrichment analysis (Fisher’s Exact Test) tool in Blast2GO software. Enrichment analysis was performed with the set of genes found uniquely in Atlantic bonito stage 30 dph and using all transcripts obtained after assembly of mixed developmental stages as reference test.
(XLSX)

Table S4 Summary of all transcripts found in the present study with a relevant role in the post-embryonic development. As described by Yufera et al. [13], selection criteria were set to genes belonging to gene families with established roles in embryonic and developmental processes.
(XLSX)
