# Supplementary material for: The Atlantic Bonito (Sarda sarda, Bloch 1793) Transcriptome and Detection of Differential Expression during Larvae Development
Source: PLoS One. 2014 Feb 4;9(2):e87744. doi: 10.1371/journal.pone.0087744 (PMC3913633; doi:10.1371/journal.pone.0087744)
Supplement: Figure S1 — Supplemental figure to figure 8 in the manuscript. Figure 8 shows only part of the 3′UTR, which differs from each other. Here the whole alignment of the cds is shown including the start and the stop codon. (DOCX) [file pone.0087744.s001.docx]

comp55305_c0_seq1 *CACCAACTGGCACAACGCACCGGTGTTGAAGATATTTAGTTATTGGTTGA*

comp55305_c0_seq2 *CACCAACTGGCACAACGCACCGGTGTTGAAGATATTTAGTTATTGGTTGA*

comp55305_c0_seq1 *AATTCCTGACTGAAAGTCATA*ATGGATGAAGAACCAGAGAGAGCCAAGCG

comp55305_c0_seq2 *AATTCCTGACTGAAAGTCATA*ATGGATGAAGAACCAGAGAGAGCCAAGCG

comp55305_c0_seq1 CTGGGAAGGGGGCTATGAGAGGACATGGGAAGTGCTAAAGGAGGACGAAT

comp55305_c0_seq2 CTGGGAAGGGGGCTATGAGAGGACATGGGAAGTGCTAAAGGAGGACGAAT

comp55305_c0_seq1 CCGGCTCACTCAAAGCCACAGTTGAGGAGATCCTGTTCCAGTCCAAAAGG

comp55305_c0_seq2 CCGGCTCACTCAAAGCCACAGTTGAGGAGATCCTGTTCCAGTCCAAAAGG

comp55305_c0_seq1 AAAAGGGTTGTAGAGAGCCATGGACAAGTGAGGCTGGGGATGATGCGTCA

comp55305_c0_seq2 AAAAGGGTTGTAGAGAGCCATGGACAAGTGAGGCTGGGGATGATGCGTCA

comp55305_c0_seq1 TCTCTATGTGGTGATTGACTGTTCAAGAAGTATGGAAGACCAAGACTTGA

comp55305_c0_seq2 TCTCTATGTGGTGATTGACTGTTCAAGAAGTATGGAAGACCAAGACTTGA

comp55305_c0_seq1 AGCCGAACCGCCTCACCTCTACTCTAAAGCTCATGGAAGCTTTTGTTGAG

comp55305_c0_seq2 AGCCGAACCGCCTCACCTCTACTCTAAAGCTCATGGAAGCTTTTGTTGAG

comp55305_c0_seq1 GAGTATTTTGACCAAAACCCCATTAGTCAGGTGGGAATCATCACTACAAA

comp55305_c0_seq2 GAGTATTTTGACCAAAACCCCATTAGTCAGGTGGGAATCATCACTACAAA

comp55305_c0_seq1 GAATAAACGAGCTGAGAAACTGACTGACCTGGCAGGGAATCCAAAGAAGC

comp55305_c0_seq2 GAATAAACGAGCTGAGAAACTGACTGACCTGGCAGGGAATCCAAAGAAGC

comp55305_c0_seq1 ACACTGCTGCTCTCAAGAAAGCAGTGGACAATGTGTGTGTAGGAGAGCCG

comp55305_c0_seq2 ACACTGCTGCTCTCAAGAAAGCAGTGGACAATGTGTGTGTAGGAGAGCCG

comp55305_c0_seq1 TCCTTGTACAACTCTCTCAGCCTGGCCATACAGACTCTCAAACACATGCC

comp55305_c0_seq2 TCCTTGTACAACTCTCTCAGCCTGGCCATACAGACTCTCAAACACATGCC

comp55305_c0_seq1 TGGACACACGAGCCGGGAGATCTTGATCATCCTCAGCAGCCTCACCACAT

comp55305_c0_seq2 TGGACACACGAGCCGGGAGATCTTGATCATCCTCAGCAGCCTCACCACAT

comp55305_c0_seq1 GTGACCCAGCCAACATCTACGAGCTGATCAAGACCCTGAAGTCTTTGAAG

comp55305_c0_seq2 GTGACCCAGCCAACATCTACGAGCTGATCAAGACCCTGAAGTCTTTGAAG

comp55305_c0_seq1 GTGCGTGTGTCTGTGATCGGCCTGTCAGCAGAGGTTCGGGTGTGCACAGT

comp55305_c0_seq2 GTGCGTGTGTCTGTGATCGGCCTGTCAGCAGAGGTTCGGGTGTGCACAGT

comp55305_c0_seq1 GTTGACCAGAGAGACAGGCGGCTCGTACCATGTCATCCTTGATGAGAGTC

comp55305_c0_seq2 GTTGACCAGAGAGACAGGCGGCTCGTACCATGTCATCCTTGATGAGAGTC

comp55305_c0_seq1 ACTTCAAAGAGCTGCTGATGCTGCATGTCAAACCTCCACCTGCCAGCTCC

comp55305_c0_seq2 ACTTCAAAGAGCTGCTGATGCTGCATGTCAAACCTCCACCTGCCAGCTCC

comp55305_c0_seq1 TCATCTGAATGCTCTTTAATACGCATGGGTTTTCCTCAGCACACCATCGC

comp55305_c0_seq2 TCATCTGAATGCTCTTTAATACGCATGGGTTTTCCTCAGCACACCATCGC

comp55305_c0_seq1 CTCTCTGACTGACCAGGATGCCAAACCTTCATTCAGCATGTCTCATTTAG

comp55305_c0_seq2 CTCTCTGACTGACCAGGATGCCAAACCTTCATTCAGCATGTCTCATTTAG

comp55305_c0_seq1 ACAGCAGCAGTGCTCCATGTCTGTCTTTGGGAGGATACTTCTGTCCACAG

comp55305_c0_seq2 ACAGCAGCAGTGCTCCATGTCTGTCTTTGGGAGGATACTTCTGTCCACAG

comp55305_c0_seq1 TGCCACGCCAAGTACACAGAGCTTCCTGTGGAGTGTAAAGTCTGTGGTTT

comp55305_c0_seq2 TGCCACGCCAAGTACACAGAGCTTCCTGTGGAGTGTAAAGTCTGTGGTTT

comp55305_c0_seq1 GACTCTGGTGTCAGCCCCTCATCTTGCCAGATCCTTCCACCACCTCTTCC

comp55305_c0_seq2 GACTCTGGTGTCAGCCCCTCATCTTGCCAGATCCTTCCACCACCTCTTCC

comp55305_c0_seq1 CCCTCCAAGCCTTTATAGAGAGTCCTGTAGAAGAGCTCCAAGGAGACAGG

comp55305_c0_seq2 CCCTCCAAGCCTTTATAGAGAGTCCTGTAGAAGAGCTCCAAGGAGACAGG

comp55305_c0_seq1 TTCTGTCAAGCATGTGAAGGGGAGCTCAAGGATAAAAGTATATTCACCTG

comp55305_c0_seq2 TTCTGTCAAGCATGTGAAGGGGAGCTCAAGGATAAAAGTATATTCACCTG

comp55305_c0_seq1 TCCATCATGTCGCCAAGTGTTCTGTGTGGAGTGTGATCTGTTCATCCATG

comp55305_c0_seq2 TCCATCATGTCGCCAAGTGTTCTGTGTGGAGTGTGATCTGTTCATCCATG

comp55305_c0_seq1 ACTCCCTGCACTGCTGCCCCTGCTGTATTCACAGTCAGAGCGCCCCCTGA

comp55305_c0_seq2 ACTCCCTGCACTGCTGCCCCTGCTGTATTCACAGTCAGAGCGCCCCCTGA

comp55305_c0_seq1 *CAGAGACTGAAACAAACCCTCTGACCATGTACCTGGGTTTCACCCCTACA*

comp55305_c0_seq2 *CAGAGACTGAAACAAACCCTCTGACCATGTACCTGGGTTTCACCCCTACA*

comp55305_c0_seq1 *TTCAGCCAATAAGGGCCACTGAACTTGTCACTGTTGTCCACCGCTGACAG*

comp55305_c0_seq2 *TTCAGCCAATAAGGGCCACTGAACTTGTCACTGTTGTCCACCGCTGACAG*

comp55305_c0_seq1 *GATGGATATGATGTACACAGGCTGTCTATGGACCAAAGGGTCATATTTCA*

comp55305_c0_seq2 *GATGGATATGATGTACACAGGCTGTCTATGGACCAAAGGGTCATATTTCA*

comp55305_c0_seq1 *AAGGTGTTGATGATTTGTAAGATATTGGATAATTACGGTTGATCCATATC*

comp55305_c0_seq2 *AAGGTGTTGATGATTTGTAAGATATTGGATAATTACGGTTGATCCATATC*

comp55305_c0_seq1 *TTTTAATTGTGGAAGCAGCAGCTGTATTCTTGCTAAAGACGGTGCTGAGT*

comp55305_c0_seq2 *TTTTAATTGTGGAAGCAGCAGCTGTATTCTTGCTAAAGACGGTGCTGAGT*

comp55305_c0_seq1 *TGGTCTGAAGCTCCTCTCTGCTTTGAAAACTGCATCTGAGCTGCTGTAAA*

comp55305_c0_seq2 *TGGTCTGAAGCTCCTCTCTGCTTTGAAAACTGCATCTGAGCTGCTGTAAA*

comp55305_c0_seq1 *TAGCACACCGCTTTATTTCACAGAAATGGTTTGTGTACATTGTTTATACA*

comp55305_c0_seq2 *TAGCACACCGCTTTATTTCACAGAAATGGTTTGTGTACATTGTTTATACA*

comp55305_c0_seq1 *GTTTTCTACTACAGTTCTTATTGTGACATGAGTTATAATAAAACTGAAAA*

comp55305_c0_seq2 *GTTTTCTACTACAGTTCTTATTGTGACATGAGTTATAATAAAACTGAAAA*

comp55305_c0_seq1 *GTGTTTGTACTGGTTGAAATTCTTTAAGTAGAATTGTTCTATTGTTAAAA*

comp55305_c0_seq2 *GTGTTTGTACTGGTTGAAATTCTTTAAGTAGAATTGTTCTATTGTTAAAA*

comp55305_c0_seq1 *CAGCCTTTACCGAATTGCTCACATTTTATACTGTCAAATCACAAATCTTT*

comp55305_c0_seq2 *CAGCCTTTACCGAATTGCTCACATTTTATACTGTCAAATCACAAATCTTT*

comp55305_c0_seq1 *GCTATGCCGAATTTAGAATTGGAAGCAGGACTGTATCTACCATTGAGAAC*

comp55305_c0_seq2 *GCTATGCCGAATTTAGAATTGGAAGCAGGACTGTATCTACCATTGAGAAC*

comp55305_c0_seq1 *ACTGAGGTTCTGCCTTTGGTGTTTGTTTCTGTGAATTTGCAGGTTTTAGC*

comp55305_c0_seq2 *ACTGAGGTTCTGCCTTTGGTGTTTGTTTCTGTGAATTTGCAGGTTTTAGC*

comp55305_c0_seq1 *AATTTGGGGTGAGTTAACATTTTACAATTACCTTTGTGGGTATTTTATAT*

comp55305_c0_seq2 *AATTTGGGGTGAGTTAACATTTTACAATTACCTTTGTGGGTATTTTATAT*

comp55305_c0_seq1 *GCTGATTCCAGTTTTTTTAGACTCACTCAATTTCAGTTTGACTATTTGTA*

comp55305_c0_seq2 *GCTGATTCCAGTTTTTTTAGACTCACTCAATTTCAGTTTGACTATTTGTA*

comp55305_c0_seq1 *GAACAAATAAATCACTATTTTCACACTAGTGCAGCTCTGGGCCTCACTGT*

comp55305_c0_seq2 *GAACAAATAAATCACTATTTTCACACTAGTGCAGCTCTGGGCCTCACTGT*

comp55305_c0_seq1 *TTAATTCACCTAATCCTGACCTGCAAACAGAAGCTTACATGTGCAAAGAC*

comp55305_c0_seq2 *TTAATTCACCTAATCCTGACCTGCAAACAGAAGCTTACATGTGCAAAGAC*

comp55305_c0_seq1 *TGTAGTGAGAATTGTGAAAAGATGGGAGGCCACTAAAAATCTCCAGTCCT*

comp55305_c0_seq2 *TGTAGTGAGAATTGTGAAAAGATGGGAGGCCACTAAAAATCTCCAGTCCT*

comp55305_c0_seq1 *GCCTGGACTGCACTGACTGGGACATTTTGGGACGAGTGACATCAGCTGAT*

comp55305_c0_seq2 *GCCTGGACTGCACTGACTGGGACATTTTGGGACGAGTGACATCAGCTGAT*

comp55305_c0_seq1 *CTGGATGAGTATGAAGACATCTGTATCATGGCAGTTTTTGTGTGGACAAT*

comp55305_c0_seq2 *CTGGATGAGTATGAAGACATCTGTATCATGGCAGTTTTTGTGTGGACAAT*

comp55305_c0_seq1 *TGCATCCCCACATTGACCCAGGTCAAGTACAGCAACAAGCCGTGGTTTGC*

comp55305_c0_seq2 *TGCATCCCCACATTGACCCAGGTCAAGTACAGCAACAAGCCGTGGTTTGC*

comp55305_c0_seq1 *TGCAGAACTCAGCCAGCTCTGTTGAGCCAGCGATGAGGCCTACAGGAGTG*

comp55305_c0_seq2 *TGCAGAACTCAGCCAGCTCTGTTGAGCCAGCGATGAGGCCTACAGGAGTG*

comp55305_c0_seq1 *GAGATTTACAGGCCGGCCAAGAACAAGCTGAAGAGAGGAATCAGAGCAGC*

comp55305_c0_seq2 *GAGATTTACAGGCCGGCCAAGAACAAGCTGAAGAGAGGAATCAGAGCAGC*

comp55305_c0_seq1 *CAAGGAGAGGCACGCTGAGAAGCTGAAGGACAGGATCTCAGCCAACAACT*

comp55305_c0_seq2 *CAAGGAGAGGCACGCTGAGAAGCTGAAGGACAGGATCTCAGCCAACAACT*

comp55305_c0_seq1 *CATCTTCAGTTTGGAAAGTCCAAGTTCGACAGACAAAGGCTTAACCTGGA*

comp55305_c0_seq2 *CATCTTCAGTTTGGAAAGTCCAAGTTCGACAGACAAAGGCTTAACCTGGA*

comp55305_c0_seq1 *CTCCCACCCGCCCCTCATTCCACATCCATCCCCACACCTCACCTTCCCCC*

comp55305_c0_seq2 *CTCCCACCCGCCCCTCATTCCACATCCATCCCCACACCTCACCTTCCCCC*

comp55305_c0_seq1 *ACTACTGGATCAACATTATCATCATTGAGTCCATCCTCGCATCATCTATC*

comp55305_c0_seq2 *ACTACTGGATCAACATTATCATCATTGAGTCCATCCTCGCATCATCTATC*

comp55305_c0_seq1 *ATTGTTTGGTAACCAAACAGGACATTCAAAAGCAAACCCCCCCAACCAAG*

comp55305_c0_seq2 *ATTGTTTGGTAACCAAACAGGACATTCAAAAGCAAACCCCCCCAACCAAG*

comp55305_c0_seq1 *ATCATCTCCAACCCATCCCACCCTGGATACCAACTCCTAGAACTTCTTCC*

comp55305_c0_seq2 *ATCATCTCCAACCCATCCCACCCTGGATACCAACTCCTAGAACTTCTTCC*

comp55305_c0_seq1 *CTCAGAAAAAGATTGAGATCCATTAAAACCTCCACCAGCAGAAACAGAAA*

comp55305_c0_seq2 *CTCAGAAAAAGATTGAGATCCATTAAAACCTCCACCAGCAGAAACAGAAA*

comp55305_c0_seq1 *TTAAACTTTTCTAATACTTGTTTATTTCATTATTATTACTACTTGACTCT*

comp55305_c0_seq2 *TTAAACTTTTCTAATACTTGTTTATTTCATTATTATTACTACTTGACTCT*

comp55305_c0_seq1 *TGTTCTATTTGTATTATTTTATGTTGCTTGTGGGAGCAAACACCAAGACA*

comp55305_c0_seq2 *TGTTCTATTTGTATTATTTTATGTTGCTTGTGGGAGCAAACACCAAGACA*

comp55305_c0_seq1 *CATTCTTTGTATGCT-GCATACTTGGCTG****ATAAA****CAGATTCTGATTCTGA*

comp55305_c0_seq2 *CCTTCTTTGTATTCTTGCATACTTGGCTA****ATAAA****CAGGTTCTGATCTCAC*

comp55305_c0_seq1 *T-GAATA---AGTTAT------GAAGGCAGA-CCTAC--GGCCATTAGTT*

comp55305_c0_seq2 *TTGAGTATATAGTTATTCTGCTGAATGAAGAGCTTACTAAAGCACCAGTG*

comp55305_c0_seq1 *CCCGCTGTTTCCTCAAATGTAAATTTTGTCAACAACAATGACTAGGCGCA*

comp55305_c0_seq2 *AC-GTTGCATGCACAACCCCAGTTCCTTGAAACCAAAGCAGCTAAATGGA*

comp55305_c0_seq1 *CCCAAGTTTGCATGCAGTCAAC-ACTTCCCCCTCTACAAAATGCTCCATC*

comp55305_c0_seq2 *AAACAGTAATCATCAATTTTATTATTTATACCTGTGCTTTTT--------*

comp55305_c0_seq1 *ATAAACAAAGATCAAC*

comp55305_c0_seq2 *----------------*

yellow: PUF family
